# Electrochemically Supported Asymmetric Synthesis on Chiral Encoded Pt and Pt–Ir Surfaces

**DOI:** 10.1021/acs.accounts.6c00499

**Published:** 2026-09-28

**Authors:** Anousha Sohail, Wanmai Srisuwanno, Sopon Butcha, Alexander Kuhn, Chularat Wattanakit

**Affiliations:** † School of Energy Science and Engineering, 423058 Vidyasirimedhi Institute of Science and Technology, Rayong 21210, Thailand; ‡ Department of Industrial Chemistry, Faculty of Science, 26682 Chiang Mai University, Chiang Mai 50200, Thailand; § Department of Chemistry, Faculty of Science and Technology, 37698 Thammasat University, Pathum Thani 12120, Thailand; ∥ University of Bordeaux, CNRS, Bordeaux INP, ISM, UMR 5255, 16 Avenue Pey Berland, Pessac 33607, France

## Abstract

In recent years, we studied the design of chiral-encoded metal surfaces via electrodeposition as a platform for heterogeneous asymmetric synthesis, where stereochemical information is intrinsically embedded within metallic architectures together with mesoporous structures. By using lyotropic liquid crystal templating and chiral molecular imprinting, it is possible to elaborate chiral-encoded mesoporous metal electrodes by electrodeposition, particularly Pt or Pt–Ir systems, with chiral recognition sites that enable ligand-free enantioselective transformations. These designer materials exhibit high surface area, efficient mass transport, and structural robustness, overcoming key limitations of traditional enantioselective catalysis such as poor recyclability and limited stability.

Our studies demonstrate that the chiral interfaces enable highly enantioselective electrosynthesis of chiral compounds, achieving up to 95% ee with excellent durability. In addition, we implemented several concepts to enhance the enantiomeric excess, for example, pulsed electrosynthesis, and self-assembled monolayer protection of chiral-imprinted metal surfaces for highly enantioselective electrosynthesis. Furthermore, we also extended the concept to heterogeneous catalysis for asymmetric synthesis of chiral molecules, in particular, pharmaceutically relevant compounds, eventually achieving up to 95% ee with excellent durability and compatibility with flow-based systems.

We also integrated these materials into autonomous systems, developing chiral metal microswimmers that combine self-propulsion with enantioselective catalysis, thus enabling self-mixing. Despite ongoing challenges in stabilizing the imprinted chiral information, the broadening of this concept to other metals, such as multimetallic and non-noble systems, offers promising solutions. Taken together, these advancements establish this approach to create chiral-encoded metal surfaces as a versatile concept for the next generation of chiral-imprinted metals for sustainable asymmetric synthesis.

## Graphical abstract

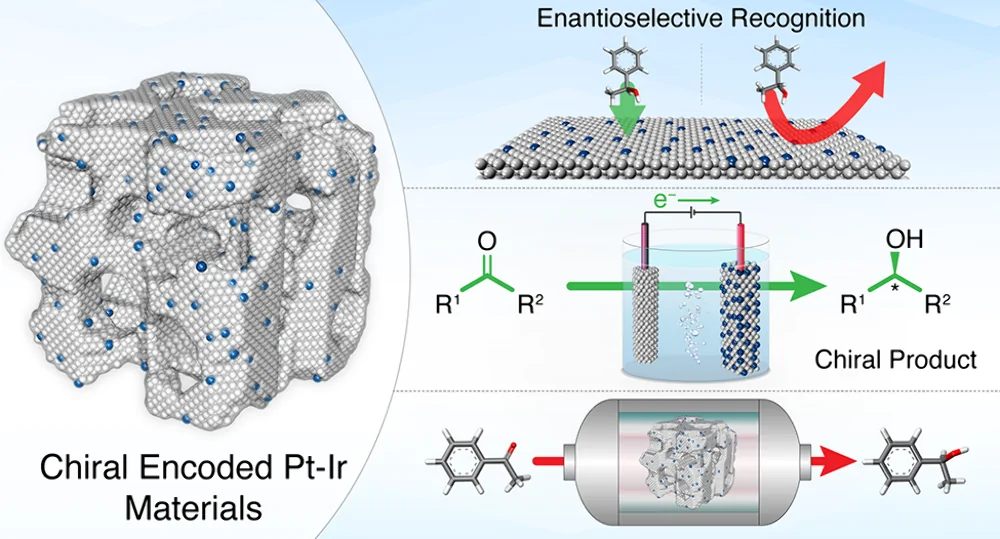

## Key References

- Butcha, S.; Assavapanumat, S.; Ittisanronnachai, S.; Lapeyre, V.; Wattanakit, C.; Kuhn, A. Nanoengineered chiral Pt–Ir alloys for high-performance enantioselective electrosynthesis. Nat. Commun. 2021, 12, 1314.10.1038/s41467-021-21603-8PMC791054233637758 ^1^ Nanostructured Pt–Ir alloys were developed as stable chiral electrocatalysts by co-electrodeposition in the presence of a chiral template and a lyotropic liquid crystal, enabling highly efficient enantioselective electrosynthesis (>95% enantiomeric excess) with improved electrochemical stability compared to the corresponding monometallic mesoporous system.
- Butcha, S.; Lapeyre, V.; Wattanakit, C.; Kuhn, A. Self-assembled monolayer protection of chiral imprinted mesoporous platinum electrodes for highly enantioselective synthesis. Chem. Sci. 2022, 13, 2339–2346.10.1039/d2sc00056cPMC886471235310499 ^2^ A synergistic strategy combining chiral-imprinted mesoporous platinum electrodes with a self-assembled monolayer of organosulfur ligands was developed to enhance enantioselectivity. This approach effectively suppresses undesired side reactions, achieving enantiomeric excesses above 90%, while significantly reducing reaction time by an order of magnitude.
- Assavapanumat, S.; Butcha, S.; Ittisanronnachai, S.; Kuhn, A.; Wattanakit, C. Heterogeneous enantioselective catalysis with chiral encoded mesoporous Pt–Ir films supported on Ni foam. Chem. Asian J. 2021, 16, 3345–3353.10.1002/asia.20210096634416087 ^3^ We reported mesoporous chiral-encoded Pt–Ir alloys supported on a porous Ni foam as heterogeneous catalysts for pulsed enantioselective synthesis of a chiral compound. The stereoselective properties originating from the chiral-imprinted sites lead to a very high enantiomeric excess (more than 80%).
- Somsri, S.; Suwankaisorn, B.; Yomthong, K.; Srisuwanno, W.; Klinyod, S.; Kuhn, A.; Wattanakit, C. Highly enantioselective synthesis of pharmaceuticals at chiral encoded metal surfaces. Chem. Eur. J. 2023, 29, e202302054.10.1002/chem.20230205437555292 ^4^ In this work, chiral-imprinted Pt–Ir nanostructured surfaces were developed as heterogeneous hydrogenation catalysts, enabling the first asymmetric synthesis of real chiral pharmaceuticals with up to 95% enantiomeric excess and good stability even using a flow reactor.
- Srisuwanno, W.; Kuhn, A.; Wattanakit, C. Towards enantioselective synthesis with autonomous chiral encoded metal-swimmers. ChemCatChem 2025, 17, e202500243.^5^ We proposed chiral-encoded metal swimmers as self-powered catalysts for enantioselective redox synthesis, using internally generated electrons from spontaneous metal oxidation to drive asymmetric reduction of prochiral precursors, while simultaneously enabling autonomous motion via hydrogen bubble propulsion.

## Introduction

Asymmetric synthesis of chiral compounds has received considerable attention, particularly in modern chemistry, because the absolute configuration of chiral molecules strongly influences their biological activities, which are crucial for the pharmaceutical industry, whereas the opposite enantiomers often exhibit markedly different physiological properties, eventually inducing toxicity.^6–8^ In addition, enantiomerically pure compounds (EPC) are essential for other applications, including agrochemicals, flavors, and functional materials.^6,7^ Traditionally, asymmetric synthesis has relied on chiral auxiliaries, organocatalysts, transition-metal complexes bearing chiral ligands, and enzymatic systems.^8–11^ Homogeneous asymmetric catalysis has revolutionized the synthesis of EPC and is widely employed in the production of pharmaceuticals and fine chemicals.^12,13^ Representative examples include asymmetric hydrogenation, transfer hydrogenation, and other transition-metal-catalyzed transformations that have been successfully implemented on preparative and industrial scales.^14,15^ Enzymatic catalysis also provides an efficient and sustainable route to enantiomerically enriched molecules owing to its excellent chemo-, regio-, and enantioselectivity.^16–18^ While these approaches exhibit high catalytic efficiency, homogeneous or enzymatic catalytic systems can face challenges in catalyst recovery, limited stability, and sustainability.^9–11^ Consequently, the development of heterogeneous catalysts with chiral features capable of inducing enantioselectivity with robustness and recyclability has become increasingly attractive.^19,20^ Considerable efforts have also been devoted to heterogeneous asymmetric catalysis through immobilization of molecular chiral catalysts on porous supports or electrode surfaces. These systems combine high enantioselectivity with improved catalyst recovery and recyclability, and have allowed for preparative-scale synthesis.^21,22^ Despite these advances, chiral-encoded metal surfaces represent a fundamentally different strategy in which chirality is embedded directly within the metallic architecture rather than introduced through surface-bound molecular modifiers.^23,24^

In this context, we provide here a comprehensive review on the design of chiral-encoded mesoporous metal surfaces and their applications in heterogeneous asymmetric synthesis of chiral compounds via both electrochemical and chemical catalytic processes.^25–28^ Unlike classic heterogeneous catalysts, which rely on chiral modifiers on solid surfaces,^29–31^ chiral-encoded electrodes incorporate stereochemical information or an intrinsically chiral feature directly into the three-dimensional architecture of the metal itself, which prevents the loss of adsorbed chiral molecules, thus preserving the chiral information.^25,26^ This concept is associated with molecular imprinting strategies developed in polymers and inorganic materials, where a molecular template directs the formation of complementary recognition sites that remain after template removal.^32–34^ Upon application to metallic systems, such an imprinting technique generates metal surfaces bearing specific chiral recognition sites or asymmetric environments, capable of enantioselective recognition and transformation of prochiral substrates to the corresponding chiral molecules.^25–28^

A breakthrough in this field is the development of chiral-imprinted mesoporous metal electrodes via an electrochemical approach (Figure 1A).^27^ In our initial work, platinum was electrodeposited on a gold-coated glass slide in the presence of a lyotropic liquid crystal phase formed by a non-ionic surfactant such as Brij^®^ 56 together with various chiral template molecules, including 3,4-dihydroxy-l-phenylalanine (DOPA) and mandelic acid.^27^ After removal of both surfactants and chiral templates, the resulting platinum retains mesoporous features and chiral recognition sites, with the non-ionic surfactant and chiral template acting as mesoporogen and chiral scaffold, respectively. Subsequently, the designer materials enable the enantioselective electroreduction of prochiral substrates, such as phenylglyoxylic acid to mandelic acid, with the product handedness dictated by the imprinted enantiomer.^25^ This first example clearly demonstrated that geometric chirality encoded within a metal matrix can induce measurable enantiomeric excess during the electrosynthesis of chiral compounds.

**Figure 1:**
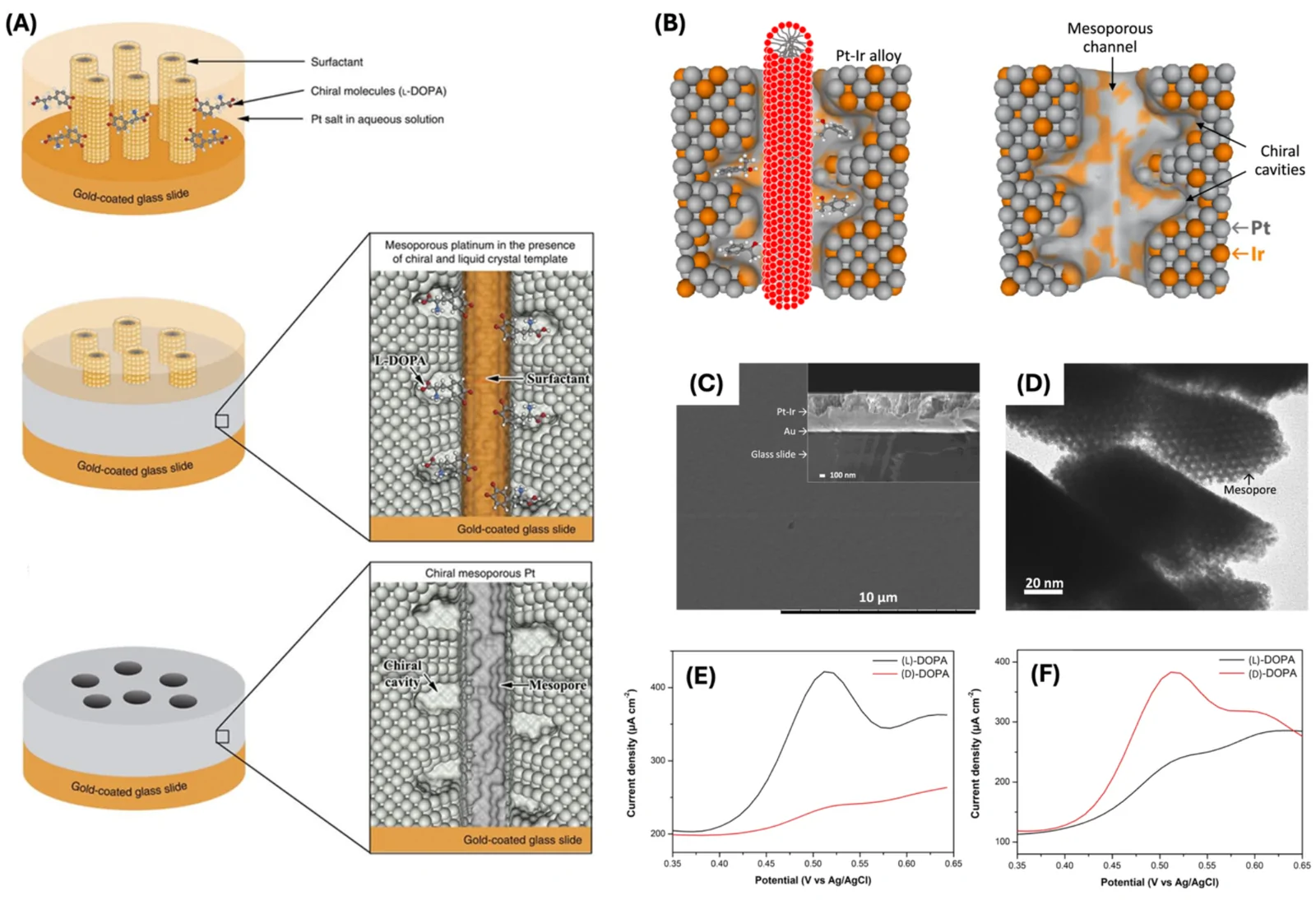
Illustration of chiral-imprinted mesoporous metal electrodes. (A) Formation of chiral mesoporous Pt via liquid-crystal templating, followed by electrodeposition and template removal. (B) Formation of chiral-encoded mesoporous Pt–Ir following the same strategy, but Ir precursor is also added. (C) and (D) SEM and TEM images of mesoporous Pt–Ir films, respectively. (E) and (F) Differential pulse voltammograms (DPV) of mesoporous Pt–Ir imprinted with L-DOPA and D-DOPA, respectively, in 4 mM L-DOPA (black) and D-DOPA (red) in 50 mM HCl as a supporting electrolyte. Reproduced from refs 1 and 27 . Available under a CC-BY 4.0 license and a CC-BY-NC-SA 3.0 license. Copyright 2021 Sopon Butcha et al. and 2014 Chularat Wattanakit et al.

The design of these materials relies critically on the formation of lyotropic liquid crystals (LLCs), which serve as soft templates to generate ordered mesoporous metal structures.^35–38^ Typically, lyotropic liquid crystals are formed when amphiphilic surfactants self-assemble in solution above the critical micelle concentration (CMC). Various mesophase structures can be formed depending on the surfactant concentration and temperature.^37,38^ The structural organization of these phases arises from the balance between hydrophilic and hydrophobic interactions and can be easily tuned by surfactant concentration, temperature, and additives, for example.^36–38^ Under certain conditions, a hexagonal LLC phase, composed of cylindrical micelles, oriented perpendicular to the electrode surface, is formed, directing the electrodeposition.^35^ In the typical procedure, metal ions are reduced around these micellar assemblies, resulting in a continuous metal network containing ordered mesochannels after surfactant removal.^35,39^

The incorporation of a chiral template within the LLC phase plays a key role in forming the chiral recognition sites embedded in the mesoporous phase.^25–27^ In fact, the chiral molecule interacts preferentially with the hydrophilic domains of the surfactant assemblies, eventually forming an oriented configuration within the mesophase.^25,40^ During electrodeposition, the metal grows within this preorganized chiral environment, effectively “locking in” stereochemical information at the nanoscale.^25,26^ Following electrodeposition, the surfactant and chiral template are removed by extensive rinsing and soaking in Milli-Q water. Previous studies verified complete template removal by monitoring the washing solutions using UV–Vis spectroscopy until no characteristic absorption of the template remained, while differential pulse voltammetry confirmed the absence of template-related oxidation signals.^27^ These control experiments demonstrate that the resulting enantioselectivity originates from the chiral-encoded metal framework rather than residual adsorbed template molecules. Once the templates are removed, chiral recognition sites remain embedded within the mesoporous metal framework.^25^ These “cavities” create asymmetric binding environments that favor interaction with one enantiomer compared to its mirror-image during subsequent electrochemical reactions, thereby inducing enantioselectivity.^25,27,28^

Figure 1A illustrates the electrodeposition process with a mixture containing a Pt salt, a lyotropic liquid crystal (surfactant), and chiral molecules (e.g., L-DOPA).^27^ The surfactant forms a hexagonal (H_1_) phase, acting as a template for mesopore formation, while the enantiomers serve as chiral templates. In the case of Pt deposition, Pt precursors are subsequently electrochemically reduced around both the liquid crystal structure and the chiral molecules, generating an ordered mesoporous framework with chiral features embedded in the mesoporous framework walls. After removal of the templates, a mesoporous Pt film with hexagonally arranged pores is obtained. As the chiral recognition sites remain within the pore walls, accessible stereochemically defined sites for enantioselective processes are provided.

Beyond providing a high surface area, the nanoscale dimensions of the mesopores can also influence the local electrochemical environment. Mesoporous metals prepared by using non-ionic surfactants such as Brij® 56 typically exhibit pore diameters of 2–5 nm with pronounced concave curvature. At this length scale, nanoconfinement and electric double-layer effects become increasingly important, particularly at low electrolyte concentrations where the double layers from opposing pore walls may overlap, thereby modifying local ion distribution, electric field, and mass transport within the pores.^41,42^ These confinement-induced effects may contribute to the sensitivity of enantioselective electrochemical reactions toward electrolyte composition and pH, reported for chiral-imprinted mesoporous metals, and should therefore be considered when interpreting catalytic performance.^3,27^ In addition to electric double-layer effects, nanoconfinement may influence molecular diffusion, adsorption behaviour, and solvent organization within the mesopores, thereby affecting reaction kinetics and selectivity. Similar confinement effects have been reported for other porous catalytic materials and may also contribute to the features of chiral-imprinted mesoporous metals.^43^

The mesoporous nature of LLC-templated electrodes offers several advantages; the high surface area and pore network can enhance mass transport of guest molecules to access the active sites more easily, which is equally important for catalysis, separation, and recognition.^35,39,44,45^ Moreover, the structural periodicity of the mesopores ensures that chiral features are distributed throughout the entire internal area of the material, distinguishing this approach from strategies involving the adsorption of chiral molecules on metal surfaces, which is limited to the outermost surface.

Following the original platinum system, the concept of chiral-encoded electrodes has also been extended to other metals.^27^ For example, the design of mesoporous chiral alloys and non-noble metals, such as Pt–Ir, Pt–Ru, and Ni, has been employed for electrochemical applications, including enantioselective discrimination and asymmetric synthesis.^1,28,46^ However, this review focuses on chiral Pt and Pt–Ir alloys, which are the most promising materials that have been developed so far, since they have exceptional structural and electrochemical stability. Accordingly, most of our examples used these materials in asymmetric electroreduction with substantial enantiomeric excess (%ee) of the desired chiral compounds.^1^ Figure 1B illustrates the elaboration of chiral-imprinted mesoporous Pt–Ir alloys. In brief, this structure was prepared via co-electrodeposition of the starting Pt and Ir precursors in the presence of lyotropic liquid crystalline phases of nonionic surfactants and chiral compounds. It should be noted that a similar strategy as the one used for the monometallic chiral Pt was applied. After surfactant and chiral template removal, Figure 1C shows scanning electron microscopy (SEM) images of chiral-imprinted mesoporous Pt–Ir electrodes, highlighting metal layers with smooth external surfaces over the whole electrode area. The high-magnification transmission electron microscopy (TEM) image of electrodeposited mesoporous Pt–Ir in Figure 1D reveals ordered mesopores resulting from the LLC templating, enabling chiral cavity distribution along the mesopore walls, which is a crucial ingredient to enhance the electrode surface area.

## Enantioselective Recognition of Chiral Compounds at Chiral Encoded Metal Surfaces

A key question for chiral inorganic electrodes is whether the introduced chirality is actually present at the electrode surface. Enantioselective recognition directly probes surface chirality by quantifying the differential interactions of the two enantiomers with the chiral-encoded surface. As can be seen in Figure 1E,F, L-DOPA-imprinted Pt–Ir alloys are globally more favorable to react with the l-enantiomer compared to the d-enantiomer, while D-DOPA-imprinted Pt–Ir alloys are globally more favorable to interact with the other enantiomer. The conceptual roots of this approach lie in molecular imprinting, where selective binding arises from structural complementarity between a template and a host matrix. Early demonstrations in inorganic systems showed that such complementarity can be preserved even after removal of the chiral template, leading to measurable enantioselective adsorption and electrochemical responses.^47,48^ These studies demonstrated that chirality could be structurally implemented into inorganic material surfaces.

Extending this concept to metallic systems, however, introduces a key challenge: surface dynamics. Unlike rigid frameworks, metal surfaces, especially ductile noble metals such as gold, can undergo restructuring, or adsorption-induced rearrangement under operating conditions. As a result, the conservation of chiral information at the outermost interface cannot be ensured. Indeed, early work on mesoporous platinum revealed that enantioselective recognition is highly sensitive to surface states, with the possibility to erase the chiral signature, particularly when applying a high positive potential.^27^ Therefore, this can lead to the destruction of chiral features either under high potential or when the electrodes are reused for several cycles. These findings underscore the necessity that chirality needs to be both encoded and structurally stabilized.

In this context, the chiral Pt–Ir alloy, engineered by co-electrodeposition in a presence of LLC and a chiral template, can recognize preferentially one enantiomer as revealed by Differential Pulsed Voltammetry (DPV). Notably, the chiral Pt–Ir alloy exhibits both high enantioselectivity and long-term electrochemical stability under quite harsh oxidative conditions.^1^

In addition to a simple readout by DPV, our recent work illustrates that the discriminating properties of these chiral metal interfaces can also be revealed via electrochemiluminescence (ECL).^49^ This approach is based on the different interactions of enantiomers, acting simultaneously as ECL co-reactants, with the chiral metal surfaces, leading to light emission with different intensity for the two enantiomers. The key finding is that the enantiomers of a probe molecule such as phenylalanine, modulate the generation of excited [Ru­(bpy)_3_]^2+^ dye, and thus the light intensity, by almost a factor of 20. The optical readout is associated with a very low background signal compared to current-based measurements, allowing subtle differences in electrochemical processes to be translated into much larger and more easily distinguishable changes in luminescence intensity. As a result, ECL can achieve significantly enhanced analytical discrimination compared to traditional electrochemical methods. This introduces an important conceptual advancement as it demonstrates that chiral-encoded metal electrodes can be coupled to secondary electrochemical processes, enabling signal amplification beyond traditional electroanalytical methods. It furthermore provides strong evidence that the encoded chirality is robust with respect to surface degradation.

Such an increased stability allowed us recently to use these electrodes also in the frame of redox cycling (RC) experiments. We elaborated an original setup composed of two chiral electrodes, facing each other to form a microchannel. Interestingly, by introducing the same chiral feature on the two parallel-oriented electrodes with opposite polarity, it is possible to achieve the preferential cyclic transformation of a specific enantiomer. We designed chiral-encoded metal layers, facing each other and constituting a thin-layer cell. By applying a suitable potential difference between these two electrodes, the enantiomer, which has a conformation corresponding to the chiral sites encoded in the pores of the anode will be oxidized. Subsequently, the generated oxidized molecules can diffuse across the microgap and get reduced back at the cathode that has been imprinted with the same chiral information. This reaction sequence repeats multiple times, depending on the gap size of the thin-layer cell, thus enhancing the faradaic current density for a given chiral analyte by more than one order of magnitude compared with a traditional electrochemical set-up and with an enantiodiscrimination efficiency reaching 90%. This chiral redox cycling (c-RC) concept opens up new perspectives for using chiral-encoded Pt–Ir alloy electrodes for chiral-sensing applications.^50^

Beyond electroanalysis, the chiral metal surfaces could also be employed for chiral separation. Using a microfluidic channel, decorated with these imprinted metals as chiral stationary phase, enantioaffinity can be fine-tuned by adjusting their potential, leading to a total separation of enantiomers.^51^

Furthermore, not only small chiral molecules, but also more complex molecular structures can be imprinted. For example, cannabinoid derivatives, including cannabidiol (CBD) and tetrahydrocannabinol (THC), were encoded in the metal phase and tested for electrochemical detection. The selectivity and sensitivity for the cannabinoid isomers were enhanced, and reusability of the sensors with insignificant sensitivity loss could be demonstrated. Detection limits as low as 1.2 μM for CBD and 1.7 μM for THC were achieved, and this analytical platform was also applied for the detection of cannabinoids in real cannabis oil samples.^52^

From a pure analytical point of view, one can therefore say that the imprinted Pt–Ir alloys exhibit excellent enantiomeric/molecular discrimination and separation, which is due to a direct interaction between the material’s nanostructure and the stereochemistry of the analytes. It is therefore reasonable to expect similar beneficial effects when using such electrodes for enantioselective synthesis.^53,54^ In other words, the enantioselective recognition discussed so far is not simply a validation step, but a central aspect for extending the functionality of these electrochemical interfaces from analytical measurements to catalysis, which will be illustrated in the following.

## Electrosynthesis of Chiral Compounds at Chiral Encoded Metal Surfaces

Electrochemical synthesis has emerged as a topic of significant interest as a green and sustainable alternative for the production of chemicals.^55–58^ From a fundamental point of view, an electrocatalytic process eliminates the need for stoichiometric redox reagents and allows precise control over driving force, enabling selective activation pathways.^55,58^ The integration of chirality directly into electrode surfaces represents a change of paradigm as the stereochemical control is no longer imparted solely by molecular catalysts, but becomes an intrinsic property of the catalytic surface itself.^1,25,28,59^ Consequently, chiral-encoded metal surfaces can play a key role in next-generation asymmetric electrosynthesis technologies.

Initially, chiral-encoded monometallic Pt and Ni electrodes were used for asymmetric electrosynthesis, although limited stability and moderate enantiomeric excess were observed.^25,28^ To address these key challenges, the rational design of chiral catalysts with improved features is required. Chiral-imprinted metallic materials have previously shown promising results as platforms for enantioselective synthesis;^59^ however, the outermost surface of such electrodes typically lacks chiral features, which can lead to non-selective interactions and undesired side reactions, lowering the enantiomeric excess. Therefore, we further developed an alternative strategy by restricting the access to the non-selective outermost electrode surface via the self-assembly of a blocking monolayer.^2^ Microcontact printing (μCP) was employed as a fast and convenient approach. For this, a thiol-containing molecule was adsorbed on a rubber stamp, which was then pressed on the outermost surfaces of the chiral-encoded electrodes. Interestingly, by fine-tuning the time allowed for the formation of a self-assembled monolayer (SAM) of such organosulfur molecules, the enantiomeric excess of chiral products can be maximized (Figure 2A). This is due to a blocking of unfavorable reaction sites, while preserving access to the chiral recognition sites, thus a significantly enhanced enantioselectivity can be achieved (>90%) when reducing e.g. the prochiral precursor acetophenone to phenyl ethanol (Figure 2B). Additionally, this allows decreasing the overall reaction time from 1 week to several hours.^2^

**Figure 2:**
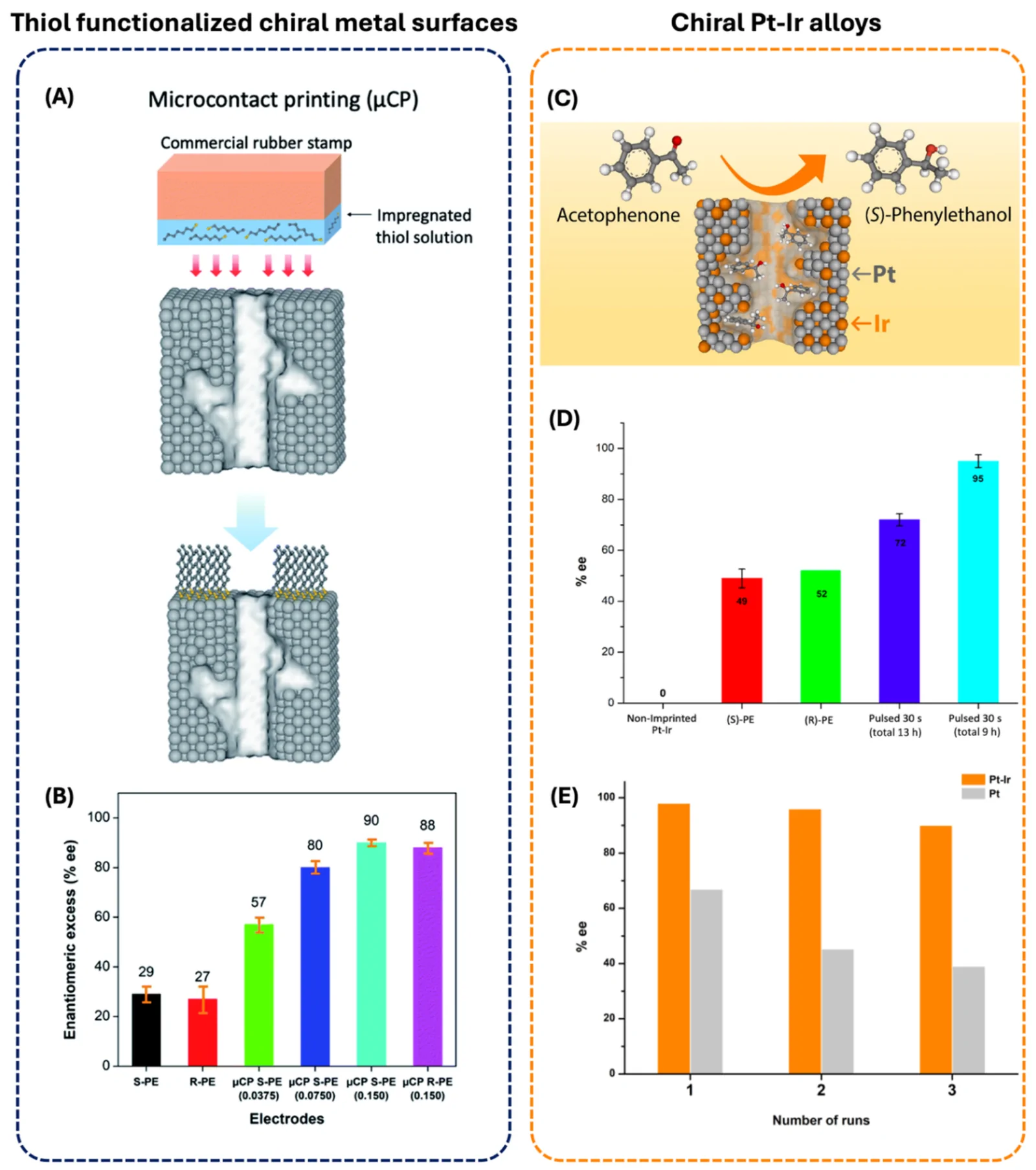
Electrosynthesis of chiral compounds with chiral-encoded metal surfaces following different strategies, including (i) thiol-functionalized chiral metal surfaces and (ii) chiral Pt–Ir alloys. (A) Formation of thiol-functionalized chiral metal surfaces via microcontact printing. (B) Histogram summarizing enantiomeric excess (%ee) values obtained after asymmetric electrosynthesis at thiol-functionalized chiral metal surfaces. (C) Electroreduction of acetophenone to 1-phenyl ethanol at chiral Pt–Ir alloys. (D) Histogram summarizing enantiomeric excess (%ee) values obtained after asymmetric electrosynthesis at chiral Pt–Ir alloys. (E) Reusability tests based on the change in %ee for chiral Pt–Ir compared to chiral Pt. Reproduced with permission from refs 1 and 2 . Available under a CC-BY 4.0 license and a CC-BY-NC 3.0 license. Copyright 2021 Sopon Butcha et al. and 2022 Sopon Butcha et al.

Apart from chiral-encoded monometallic materials, imprinted Pt–Ir has emerged as a powerful platform for the stereoselective electrosynthesis of enantiomerically enriched compounds due to its superior electrochemical stability and activity, generated by only a small portion of Ir (approximately 10-15 at. %) in the alloy, which alters the electronic structure of Pt, resulting in an increased hardness. The recognition sites enable selective molecular adsorption and further asymmetric induction during the electroreduction of aryl alkyl ketones to alcohols, as shown in Figure 2C. The proposed process involves the adsorption of prochiral substrates onto imprinted sites, followed by proton-coupled electron transfer within a stereochemically confined environment, thereby favoring the formation of one enantiomer over the other with >95% enantiomeric excess, shown in Figure 2D. It is also worth noting that the chiral Pt–Ir alloys can be reused for several catalytic cycles with an insignificant decrease in enantiomeric excess when compared to the previously employed monometallic Pt platform (Figure 2E).^1^ Apart from electrochemical enantioselective synthesis, the Pt–Ir alloys can also be used in the frame of asymmetric hydrogenation. For example, chiral Pt–Ir supported on Ni foam can be used as a heterogeneous catalyst for the asymmetric synthesis of phenyl ethanol via hydrogenation of acetophenone as a model compound, which will be discussed in the following.^3^

Overall, the remarkable enhancement in both catalytic activity and stability of chiral-encoded Pt–Ir alloys effectively addresses key limitations that have previously hindered the practical use of imprinted metal electrodes. Beyond their fundamental scientific significance, these developments highlight the potential of chiral Pt–Ir alloys for more practical implementations as discussed in the following. Recently, privileged chiral catalysts have been the focus of extensive research efforts, leading to various innovative developments. The integration of chiral scaffolds into the metal domain is therefore promising. Considering the concept of the molecular imprinting approach, the chiral template interacts with the nonionic surfactant assembled in a columnar mesostructure through intermolecular hydrogen bonding during metal growth.^27^ This allows the formation of chiral cavities inside the mesoporous structure of metals after template removal. Thus, the application of chiral molecules as a template has gained considerable attention, which is expected to further push the boundaries of this field.^60^

## Enantioselective Synthesis of Chiral Pharmaceuticals

Recent developments in transition-metal-catalyzed asymmetric synthesis further highlight the versatility of molecular asymmetric catalysts.^61^ For instance, transition-metal catalysis remains foundational, which enables high enantioselectivity in the synthesis of chiral pharmaceutical compounds. For instance, iridium-catalyzed asymmetric C–H borylation of α-all-carbon substituted 2,2-dimethyl amides was reported using a rationally designed chiral bidentate boryl ligand, leading to a range of acyclic all-carbon quaternary stereocenters, which serve as a crucial unit in various pharmaceuticals.^62^ In addition, palladium has been considered as a promising alternative for enantioselective C–H olefination, yielding meta-disubstituted paracyclophane derivatives which are applicable as anticancer agents.^63^ Molecular imprinted metals as heterogeneous catalysts provide stereochemical information directly in the metallic architectures, enabling recyclability and separation of the catalyst from the enantiomeric products.^1^ To extend the scope of using encoded metals as heterogeneous catalysts, we have recently reported proof-of-principle experiments for the asymmetric hydrogenation of aromatic ketones using chiral-encoded Pt–Ir alloys supported on three-dimensional high-surface-area nickel foam (3D Ni foam).^3^ The 3D Ni foam was chosen to facilitate the diffusion of educts and products. As previously, the mesoporous chiral-encoded Pt–Ir alloys were obtained by electrodeposition on porous nickel foam in the simultaneous presence of phenyl ethanol, serving as a chiral model template, and non-ionic surfactants. Interestingly, an enantiomeric excess of approximately 85% could be achieved when using pulsed hydrogenation of acetophenone. The synergy between the 3D porous Ni foam and the mesoporous Pt–Ir alloys allows maintaining high enantioselectivity for multiple catalytic cycles.^3^ This opens up interesting perspectives for the synthesis of chiral products from prochiral precursors even without applied potential.

Apart from the above-mentioned example, heterogeneous catalysis using chiral-encoded metals has been employed to synthesize enantioenriched pharmaceuticals.^4^ The well-defined chiral features in the metal domain lead to high stereoselectivity for the hydrogenation of 3’,4’-dihydroxy-2-(methylamino)­acetophenone (adrenalone) as a prochiral precursor, generating epinephrine (EP), serving as an emergency medical treatment such as for anaphylaxis, laryngeal edema, and bronchospasm (Figure 3A). The Pt–Ir alloys imprinted with (L)-EP showed preferential synthesis of (L)-EP, with a very high enantioselectivity (95%ee) with approximately 4% product yield when pulsed hydrogenation was applied (Figure 3B,C). Under these conditions, adrenalone was allowed sufficient time to adsorb on chiral recognition sites before hydrogen was introduced into the system, while the produced EP had enough time to desorb from these recognition sites. As expected, a specular result was obtained for the experiments carried out with (D)-EP-imprinted Pt–Ir electrodes, favoring the production of (D)-EP (Figure 3C). The chiral features encoded in the metal domain were stable, evidenced by a high enantiomeric excess of up to 90% over multiple cycles (Figure 3D). During enantioselective transformation, molecular chiral cavities influence the spatial orientation of a prochiral precursor during the transition state of the enantioselective synthesis, eventually leading to the formation of the chiral product, which is identical to the structure of the cavities. However, a certain mismatch between the chiral template and a desired product can be allowed. Typically, the concept of molecular imprinting in asymmetric catalysis strongly depends on the chiral center of the molecule and the functional group, where the reaction occurs. For this, a similar configuration of the functional group at the chiral center compared to the one of the desired product can be used as a template. One example is the asymmetric synthesis of epinephrine (EP) via hydrogenation of adrenalone using phenyl ethanol-imprinted Pt–Ir alloys. The presence of the OH group at the chiral center in the structure of phenyl ethanol enables controlling the spatial orientation of adrenalone during the enantioselective hydrogenation to epinephrine, leading to more than 60% enantiomeric excess. Notably, the catalytic performance, in particular the product yield is restricted by the limited active surface and the mass transport of educts and products to and from the chiral-imprinted sites within the pores. However, this work established that nanoscale surface imprinting is able to translate molecular chirality into durable heterogeneous catalytic interfaces suitable for pharmaceutical synthesis.^4^ Although chiral-encoded metal surfaces have shown promising proof-of-concept results, important challenges remain in comparison with established homogeneous and immobilized heterogeneous catalysts, particularly regarding substrate scope, catalytic performance, and scalability. Future work should focus on improving catalyst efficiency, understanding reaction mechanisms, and enabling larger-scale applications.

**Figure 3:**
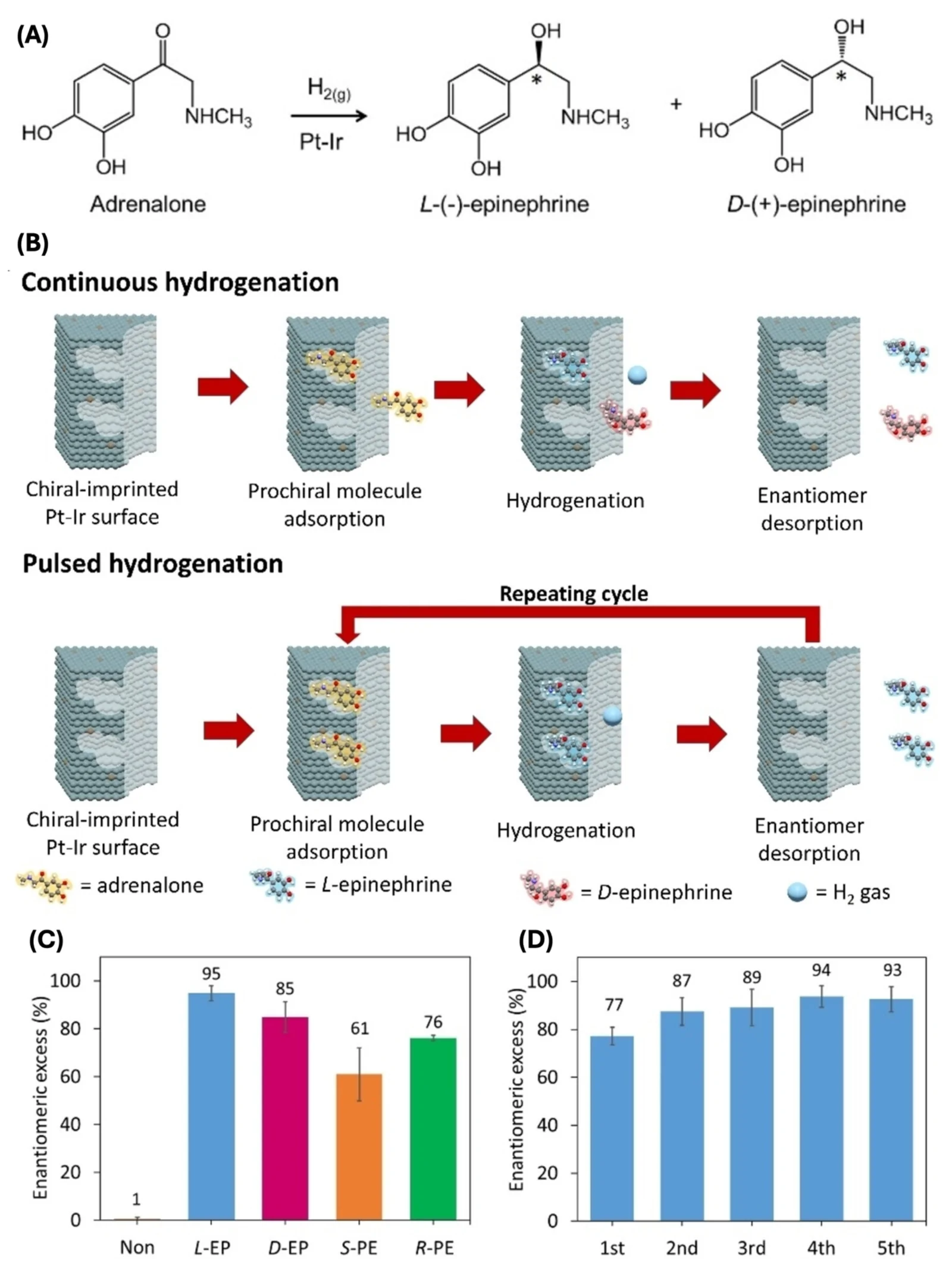
Asymmetric hydrogenation of adrenalone to epinephrine on chiral-imprinted Pt–Ir alloy surfaces. (A) Reaction scheme of hydrogenation of adrenalone to epinephrine. (B) Two catalytic approaches using continuous or pulsed hydrogenation on Pt–Ir surfaces containing chiral molecular recognition sites. (C) Histograms showing the enantiomeric excess (ee) of epinephrine obtained by using the mesoporous Pt–Ir alloys under different imprinting conditions. (D) Histograms showing the enantiomeric excess (ee) of epinephrine obtained by using the mesoporous Pt–Ir alloys imprinted with (l)-epinephrine for successive cycles of operation. Reproduced with permission from ref 4. Copyright 2023 Wiley-VCH.

## Chiral Encoded Metal Swimmers for Autonomous Enantioselective Synthesis

Although the application of chiral-imprinted metals for producing chiral molecules has been validated by the above-mentioned proof-of-principle experiments, the rather low production rate of the discussed catalytic processes needs to be addressed. One limiting factor is the mass transport of the prochiral precursor toward the surface of the chiral catalyst or vice versa as the electrode constitutes only a two-dimensional reaction space. Recently, the introduction of convection at the macro-, micro-, and nano-scale has been proposed to overcome this limitation for numerous applications, ranging from sensing to environmental remediation and cargo delivery.^64–66^ Distinct propulsion mechanisms have been developed using a variety of strategies such as self-electrophoresis,^67^ diffusiophoresis,^68^ magnetic,^69,70^ light-driven,^71^ ultrasound,^72^ and electric field propulsion,^73^ leading to devices combining selective chemical features coupled with motion.^74–76^ One specific example is based on redox-active autonomous chiral microswimmers, enabling highly efficient asymmetric synthesis of a chiral product from a prochiral starting compound. This work established improved mass transport by convection that takes place along the entire surface of the microswimmers, while mechanical stirring is not efficient enough to improve mass transport everywhere on the active surface.^77^ In this context, the key idea is to replace conventional 2D wired electrodes by redox-active microswimmers bearing chiral metal layers such as Pt, Ir, or Au, supported on reactive metals like Zn and Fe, which allows mimicking a pseudo 3D reaction space if enough swimmers are exposed to the solution.

The mesoporous chiral-encoded metals were turned into a self-propelled swimmer, serving as an emerging class of mobile catalyst systems. Recently, we have reported a proof-of-concept experiment for the enantioselective redox synthesis of a chiral product via the stereoselective reduction of a prochiral compound (Figure 4).^5^ The chiral-imprinted swimmers have been elaborated by the spontaneous reduction of Pt and Ir precursors or Ni salts on the surface of zinc (Zn), serving as a reducing agent, in the simultaneous presence of a non-ionic surfactant and a chiral template, which direct the formation of mesoporous channels and chiral recognition sites, respectively. The breaking of symmetry for the rod-like swimmer allowed generating the object’s motion, and a simultaneous production of chiral products as illustrated in Figure 4A,B. The self-propelled chiral-encoded swimmer enables enantioselective synthesis of phenyl ethanol from acetophenone in an acid solution by utilizing its internal thermodynamic driving force. Under this condition, the spontaneous oxidation of Zn at the core shuttles a fraction of electrons to the chiral-encoded metal located at the shell, thus triggering the enantioselective reduction of prochiral acetophenone, leading to the asymmetric formation of the corresponding phenyl ethanol enantiomer (Figure 4D,E). The remaining electrons are consumed for proton reduction, thus allowing an autonomous propulsion of the swimmers via hydrogen bubbles due to an asymmetric surface coating of the Zn wire (Figure 4C). By optimizing the reaction conditions to avoid undesired reactions, in particular the formation of pinacol, an enhancement of enantiomeric excess could be achieved. Even though only a low enantioselectivity is obtained, these proof-of-principle experiments pave the way for scalable redox synthesis of a chiral compound from a prochiral precursor. Indeed, it is possible to further enhance the efficiency of chiral synthesis, e.g. by multiplying the number of swimmers in contact with the prochiral precursor. With respect to future applications, still other challenges remain, including an improvement of the catalytic activity and catalyst stability under continuous motion, as well as expanding the reaction scope beyond model substrates. Nonetheless, chiral-encoded swimmers represent a promising strategy for autonomous enantioselective catalysis that integrates heterogeneous catalysis, electrochemistry, and active matter.^77,78^ Apart from transforming classic Pt–Ir electrodes into self-propelled Pt–Ir-swimmers, the development of an electrocatalytic cyclic deracemization process using chiral-imprinted metal surfaces has also been the focus of research efforts, allowing the production of a pure enantiomer from a racemic mixture.

**Figure 4:**
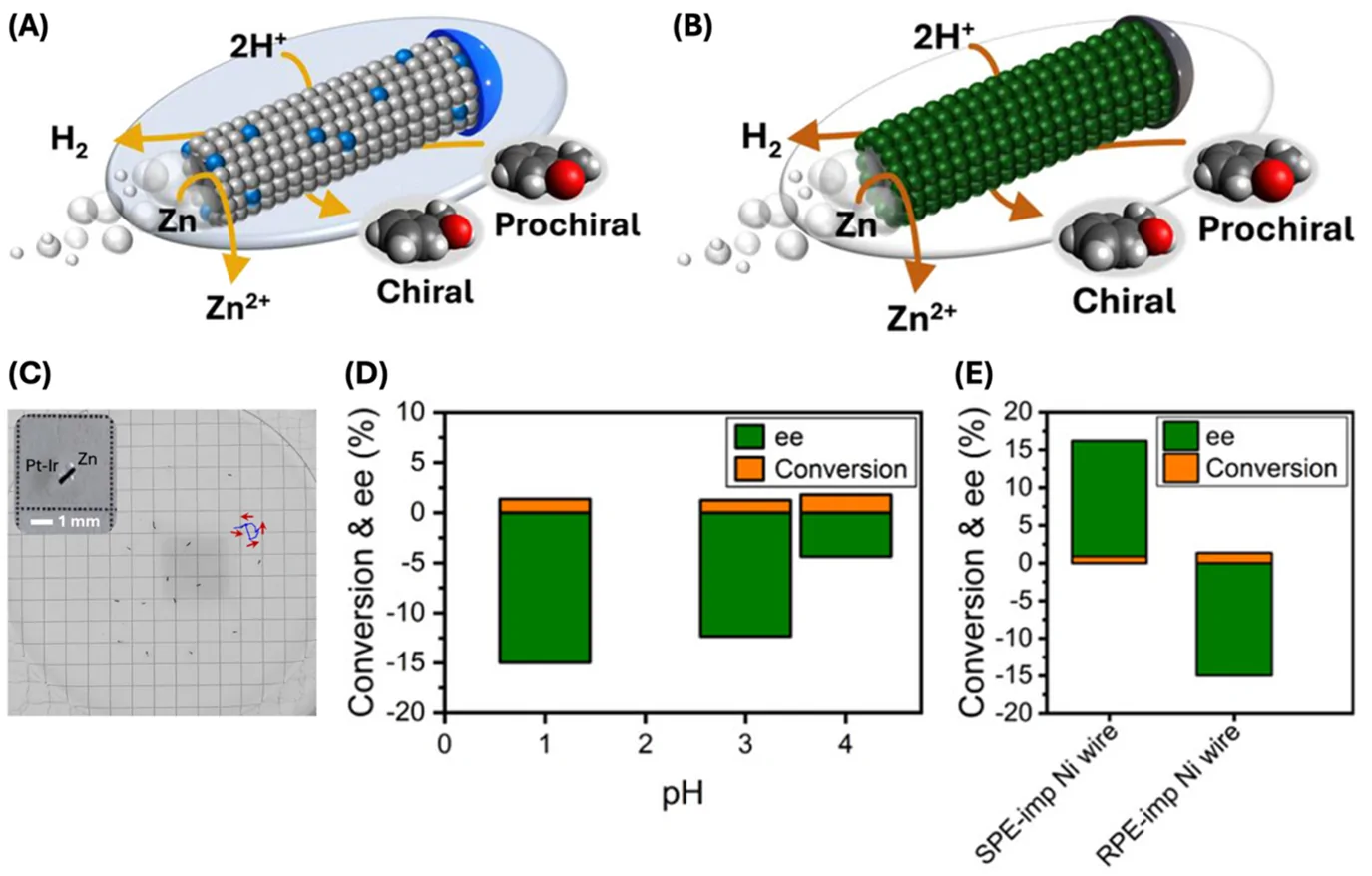
Chiral self-propelled metal swimmers for asymmetric synthesis. (A) Schematic of a chiral-encoded Pt–Ir–Zn swimmer with a Pt–Ir shell and Zn core. (B) Schematic of a chiral-encoded Ni–Zn wire with a Ni shell and Zn core. (C) Representative trajectory of a Pt–Ir–Zn swimmer in 30 mM acetophenone and 0.15 M H_2_SO_4_. (D) Influence of proton concentration on phenyl ethanol formation using (*R*)-phenyl ethanol-imprinted Ni–Zn wires. (E) Effect of chiral templates on asymmetric phenyl ethanol synthesis. Reproduced with permission from ref 5. Copyright 2025 Wiley-VCH.

## Conclusion and Outlook

In this account, we have discussed the development of chiral-encoded metal surfaces, in particular Pt and Pt–Ir, which have emerged as a transformative platform for heterogeneous asymmetric synthesis, enabling stereocontrol to be embedded directly within metallic architectures through templated electrodeposition. Unlike conventional systems, these materials integrate chiral features in mesoporous frameworks of metals and alloys, formed via lyotropic liquid crystal templating, generating stable and accessible chiral recognition sites that drive enantioselective transformations. Advances in Pt–Ir and related systems demonstrate high enantiomeric excesses (up to >95%) in the asymmetric synthesis of chiral model products and even pharmaceutically relevant compounds, while also offering robustness, recyclability, and compatibility with sustainable processes.

Despite these significant advances, several challenges remain to be addressed. For example, a deeper understanding of the charge transfer occurring on the chiral metal surfaces is necessary since these materials are mainly used for electrochemical transformations. In addition, expanding the scope of applications and improving long-term stability of the catalysts should also be a focus of further research. Multimetallic catalysts should be further explored, in particular based on non-noble metals, as they are expected to be promising alternative candidates compared to the quite expensive noble-metal catalysts. Additional studies should explore in more detail the concept of self-propelling catalyst particles, especially the effect of their composition, size and shape in order to optimize the autonomous motion and the associated enantioselectivity. In addition, future work should focus on improving catalyst efficiency and enabling larger-scale applications. As a starting point of these different optimization challenges, this account illustrates the current state-of-the-art with respect to the design of chiral-imprinted metal surfaces for a variety of potential applications ranging from analysis, to separation, and catalysis. This should constitute a strong motivation to further address key challenges, particularly those associated with scale-up and high yield synthesis.
